# 738. Spatiotemporal Patterns of Antimicrobial Resistance of Outpatient *Staphylococcus aureus* Isolates in the United States, 2010-2019

**DOI:** 10.1093/ofid/ofad500.799

**Published:** 2023-11-27

**Authors:** Matthew Smith, Margaret Carrel, Qianyi Shi, Shinya Hasegawa, Gosia Clore, Austin Tang, Eli N Perencevich, Michihiko Goto

**Affiliations:** University of Iowa, Iowa City, Iowa; Univeristy of Iowa, Iowa City, Iowa; Iowa City VA Health System, Iowa City, Iowa; University of Iowa Carver College of Medicine, Iowa City, Iowa; University of Iowa, Iowa City, Iowa; University of Iowa, Iowa City, Iowa; University of Iowa/Iowa City VAMC, Iowa City, Iowa; University of Iowa/Iowa City VAMC, Iowa City, Iowa

## Abstract

**Background:**

Oral non-beta-lactam antibiotics are commonly used for empiric therapy of *Staphylococcus aureus* infections, especially in outpatient settings. However, little is known about geographic heterogeneity in the prevalence and temporal trends of antibiotic resistance among different regions in the US. We aimed to characterize the spatiotemporal patterns of drug-resistance prevalence of *S. aureus* using the nationwide surveillance data from the Veterans Health Administration (VHA) system.

**Methods:**

Utilizing a dataset of 383,514 *S. aureus* isolates obtained in outpatient settings in the VHA from 2010-2019, we explored the spatiotemporal variation of *S aureus* resistance to clindamycin, tetracyclines, trimethoprim-sulfamethoxazole, and macrolides, stratified by methicillin-resistant *S. aureus* (MRSA) and methicillin-sensitive *S. aureus* (MSSA), and subdivided by regions of the United States (Northeast, Midwest, South, and West).

**Results:**

Over the ten-year study period there was a national decrease in the proportion of *S. aureus* isolates which were MRSA, from 54% to 39% (Figure 1). Amongst MRSA isolates (Figure 2, panels A-D), we observed stability of clindamycin resistance (from 24.5% to 31.1%), an increase in tetracycline resistance (from 3.9% to 13.1%), an increase in trimethoprim-sulfamethoxazole resistance (from 2.7% to 9.3%), and a decrease in macrolide resistance (from 72% to 60%). For MSSA (Figure 2, panels E-H), we observed relative stability of resistance over time for all four drug classes. Regional analysis (Figure 3) demonstrated that the Northeastern US had slightly higher rates of clindamycin resistance than other regions but lower rates of tetracycline resistance, while the South had notably higher rates of trimethoprim-sulfamethoxazole resistance than other regions, particularly amongst MRSA isolates.

Figure 1

**Figure:**
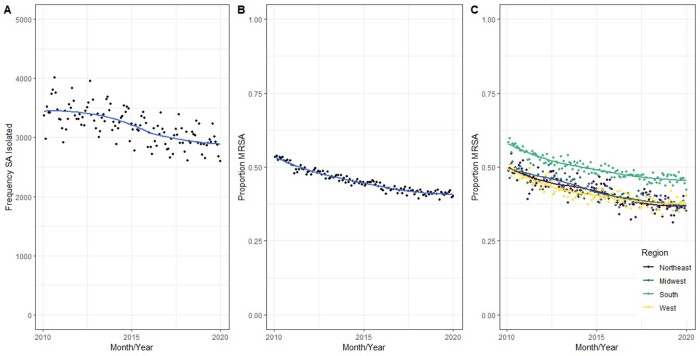

Frequency of S. aureus isolates by year (A), proportion of S. aureus isolates resistant to methicillin (B), and proportion of S. aureus isolates resistant to methicillin by region (C)

Figure 2

**Figure:**
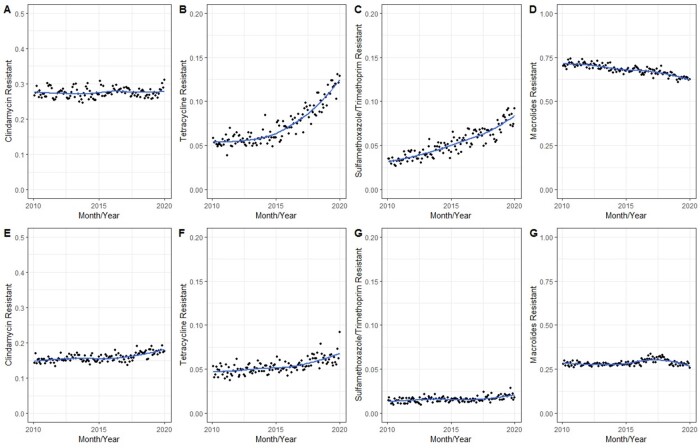

Proportion of S aureus isolates resistant to Clindamycin, Tetracyclines, Trimethoprim-Sulfamethoxazole, and Macrolides, stratified by MRSA (panels A-D) and MSSA (panels E-H)

Figure 3

**Figure:**
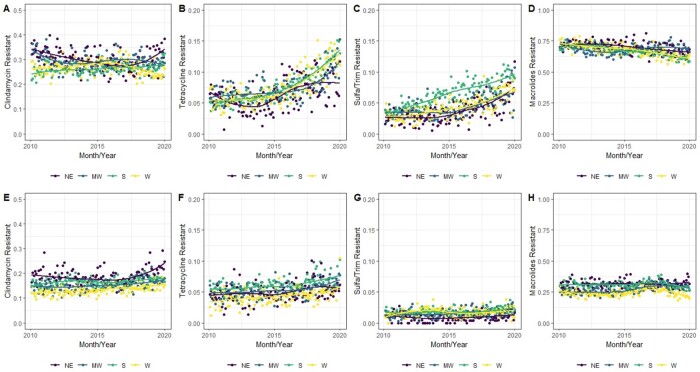

Proportion of S aureus isolates resistant to Clindamycin, Tetracyclines, Trimethoprim-Sulfamethoxazole, and Macrolides, stratified by MRSA (panels A-D) and MSSA (panels E-H), subdivided by region

**Conclusion:**

Even though the prevalence of MRSA is decreasing nationally, there are variable levels of co-resistance to non-beta-lactam antibiotics amongst *S. aureus* isolates. There was some regional variability in co-resistance amongst MRSA isolates, but less so for MSSA isolates. Further studies are needed to better understand which biological mechanisms mediate these differences.

**Disclosures:**

**Michihiko Goto, MD MSCI**, Merck & Co.: Grant/Research Support

